# Association Between Health Insurance Literacy and Avoidance of Health Care Services Owing to Cost

**DOI:** 10.1001/jamanetworkopen.2018.4796

**Published:** 2018-11-16

**Authors:** Renuka Tipirneni, Mary C. Politi, Jeffrey T. Kullgren, Edith C. Kieffer, Susan D. Goold, Aaron M. Scherer

**Affiliations:** 1 Institute for Healthcare Policy & Innovation, University of Michigan, Ann Arbor; 2 Division of General Medicine, Department of Internal Medicine, University of Michigan, Ann Arbor; 3 Division of Public Health Sciences, Department of Surgery, Washington University School of Medicine in St Louis, St Louis, Missouri; 4 Veterans Affairs Center for Clinical Management Research, Veterans Affairs Ann Arbor Healthcare System, Ann Arbor, Michigan; 5 Center for Bioethics and Social Sciences in Medicine, University of Michigan, Ann Arbor; 6 School of Social Work, University of Michigan, Ann Arbor; 7 Department of Health Management and Policy, School of Public Health, University of Michigan, Ann Arbor; 8 Department of Internal Medicine, University of Iowa Carver College of Medicine, Iowa City

## Abstract

**Importance:**

Navigating health insurance and health care choices requires considerable health insurance literacy. Although recommended preventive services are exempt from out-of-pocket costs under the Affordable Care Act, many people may remain unaware of this provision and its effect on their required payment. Little is known about the association between individuals’ health insurance literacy and their use of preventive or nonpreventive health care services.

**Objective:**

To assess the association between health insurance literacy and self-reported avoidance of health care services owing to cost.

**Design, Setting, and Participants:**

In this survey study, a US national, geographically diverse, nonprobability sample of 506 US residents aged 18 years or older with current health insurance coverage was recruited to participate in an online survey between February 22 and 23, 2016.

**Main Outcomes and Measures:**

The validated 21-item Health Insurance Literacy Measure (HILM) assessed individuals’ self-rated confidence in selecting and using health insurance (score range, 0-84, with higher scores indicating greater levels of health insurance literacy). Dependent variables included delayed or foregone preventive and nonpreventive services in the past 12 months owing to perceived costs, and preventive and nonpreventive use of services. Covariates included age, sex, race/ethnicity, income, educational level, high-deductible health insurance plan, health literacy, numeracy, and chronic health conditions. Analyses included descriptive statistics and bivariate and multivariable logistic regression.

**Results:**

A total of 506 of 511 participants who began the survey completed it (participation rate, 99.0%). Of the 506 participants, 339 (67.0%) were younger than 35 years (mean [SD] age, 34 [10.4] years), 228 (45.1%) were women, 406 of 504 who reported race (80.6%) were white, and 245 (48.4%) attended college for 4 or more years. A total of 228 participants (45.1%) had 1 or more chronic health condition, 361 of 500 (72.2%) who responded to the survey item had seen a physician in the outpatient setting in the past 12 months, and 446 of the 501 (89.0%) who responded to the survey item had their health insurance plan for 12 or more months. One hundred fifty respondents (29.6%) reported having delayed or foregone care because of cost. The mean (SD) HILM score was 63.5 (12.3). In multivariable logistic regression, each 12-point increase in HILM score was associated with a lower likelihood of both delayed or foregone preventive care (adjusted odds ratio [aOR], 0.61; 95% CI, 0.48-0.78) and delayed or foregone nonpreventive care (aOR, 0.71; 95% CI, 0.55-0.91).

**Conclusions and Relevance:**

This study’s findings suggest that lower health insurance literacy may be associated with greater avoidance of both preventive and nonpreventive services. It appears that to improve appropriate use of recommended health care services, including preventive health services, clinicians, health plans, and policymakers may need to communicate health insurance concepts in accessible ways regardless of individuals’ health insurance literacy. Plain language communication may be able to improve patients’ understanding of services exempt from out-of-pocket costs.

## Key Points

**Question:**

What is the association between health insurance literacy and avoidance of health care services owing to cost?

**Findings:**

In this US national survey study of 506 insured adults, 29.6% reported having delayed or foregone care because of cost. Higher health insurance literacy was associated with a lower likelihood of delayed or foregone care owing to cost for both preventive and nonpreventive care.

**Meaning:**

These findings suggest that to improve appropriate use of recommended health care services, including preventive health services, clinicians and policymakers may need to adopt communication strategies that make health insurance concepts accessible to individuals regardless of health insurance literacy and improve consumers’ understanding of services exempt from out-of-pocket costs.

## Teaser

This survey study examines the association between delayed or foregone preventive and nonpreventive health care service use and the level of understanding of health insurance coverage among US adults.

## Introduction

Few Americans have a complete understanding of health insurance terms and details. In 2 recent national studies, only 4% to 14% of individuals were able to respond correctly to a set of questions assessing basic understanding of health insurance.^1,2^ This lack of understanding was most pronounced among low socioeconomic status, racial/ethnic minority, older, or previously uninsured populations who often have high health needs necessitating use of health care services.^1,2,3,4,5^ Knowledge and application of health insurance concepts (ie, health insurance literacy) have become increasingly important for all Americans as a greater number of health plans have complex cost-sharing features that can change annually, and many previously uninsured individuals may be newly accessing health care after coverage expansion through the Affordable Care Act (ACA).^6,7^

Despite this need, there is an overall dearth of studies examining the association between individuals’ health insurance literacy and their health and health care use.^8^ Although there is a broad amount of literature on the outcome of general health literacy (defined as individuals’ ability to understand health information needed to make health care decisions), studies of health insurance literacy are limited.^9,10^ Most prior studies on this topic have focused on consumers’ ability to select a health insurance plan.^11,12,13,14,15,16,17,18^ Less is known about how patients navigate and use health insurance after obtaining insurance. Understanding insurance cost-sharing features, including deductibles, copayments, and coinsurance, may facilitate appropriate care-seeking and may help to prevent individuals from delaying or avoiding needed care owing to costs. In 1 study of Medicare beneficiaries, those who were less familiar with the details of their Medicare coverage were more likely to delay care because of cost, have multiple emergency department visits, and rate their overall health as poorer.^19^ Other studies have found that aspects of low health insurance literacy—including lack of knowledge of drug coverage and difficulty estimating copayments—were associated with medication nonadherence^20^ and delays or avoidance of outpatient care.^21^

Understanding health insurance coverage may be particularly important for patients’ decisions about whether to seek preventive services. Preventive care may be perceived as discretionary because it does not address acute care needs or distressing symptoms. Under the ACA, services such as cancer screenings and vaccinations that are recommended by the US Preventive Services Task Force, Advisory Committee on Immunization Practices, Health Resources and Services Administration, or National Academy of Medicine are considered exempt from out-of-pocket payments by consumers.^22^ Yet many individuals may remain unaware of this cost-sharing exemption, worry about possible out-of-pocket costs, and consequently delay preventive care.^23,24^

This study sought to determine the association between health insurance literacy and avoidance of health care services owing to perceived out-of-pocket costs. Specifically, we aimed to assess delay or avoidance of preventive and nonpreventive health services.

## Methods

### Study Design and Participants

We recruited a national, geographically diverse, nonprobability sample to participate in an online survey using Amazon’s Mechanical Turk (MTurk), an online source of participants used frequently in social science research.^25,26^ MTurk allows researchers to post online studies that individuals may complete for a small monetary payment. MTurk participants are more representative of the US population than in-person convenience samples,^27^ and the method has been demonstrated to provide similar results for psychological and political outcomes as benchmark national samples.^28^ The study was deemed exempt by the University of Michigan Institutional Review Board with waiver of informed consent. The participants were compensated $1.50 after completing the survey. This study followed the American Association for Public Opinion Research (AAPOR) reporting guideline.

To be included in the study, participants had to be US residents aged 18 years or older with health insurance at the time of the survey. After recruitment and electronic consent, participants completed an online Qualtrics survey, available through MTurk, between February 22 and 23, 2016; the data were accessed for analysis from the site on February 23, 2016.

### Measures

#### Delayed or Foregone Care

Our primary dependent variable was delayed or foregone care owing to perceived costs. Participants were asked, “During the past 12 months, have you delayed seeking medical care because of worry about the cost?” and “During the past 12 months, was there any time when you needed medical care but did not get it because you could not afford it?”^29^ Those who responded yes to either item were asked whether they delayed or went without care for several examples of preventive services (ie, annual routine physical examination; blood test for cholesterol level check; influenza vaccination; colon cancer screening test, such as a stool test, colonoscopy, or sigmoidoscopy; routine mammogram; Papanicolaou testing); response options included yes, no, or not applicable. These are services that would be exempt from cost sharing under the ACA.

Participants were also asked whether they delayed or went without care for several examples of nonpreventive services (visit to the physician for cough, radiograph for broken bone, magnetic resonance imaging for muscle or joint pain). These services were selected as representative types of urgent or nonroutine care that participants may have experienced. Participants could respond yes, no, or that the service was not applicable to them. We dichotomized these items as avoidance vs no avoidance of each service and analyzed aggregate measures for delaying or foregoing any preventive care and any nonpreventive care. Participant eligibility for health care services was defined as cholesterol level check (men aged ≥35 years and women aged ≥45 years; n = 133), colon cancer screening test (participants aged 50-75 years; n = 57), mammogram (women aged 21-65 years; n = 35), Papanicolaou test (women aged 21-65 years; n = 224), and all other health care services (all participants; N = 506).

#### Use of Preventive and Nonpreventive Services

Our secondary dependent variable was participants’ self-reported use of selected common preventive (influenza vaccination in the past 12 months, cholesterol level check in the past 5 years)^30^ and nonpreventive (emergency department visit, hospital admission) services.^29^ As above, we considered these items individually and in aggregate for any preventive or any nonpreventive health services use.

#### Health Insurance Literacy, Health Literacy, and Numeracy

Our key independent variable was health insurance literacy, as assessed by the validated 21-item Health Insurance Literacy Measure (HILM), which is, to our knowledge, the only currently validated measure of health insurance literacy for the general population.^8^ Similar to other subjective measures that have been demonstrated to be important in medical decision making (eg, subjective numeracy and health literacy),^31,32^ an advantage of the HILM is that, although it is correlated with objective measures of health insurance–related knowledge and skills, it measures motivation to engage in health insurance–related behaviors (eg, information seeking, document literacy), in addition to self-reported ability and knowledge.^8^ We assigned a score to each of the 21 items based on confidence in health insurance understanding and navigation (score of 1 was assigned to a response of not at all confident; 2, slightly confident; 3, moderately confident; and 4, very confident) and summed scores for individual items to create a scaled score with a range of 0 to 84 (eFigure in the Supplement indicates variable distribution). Higher HILM scores indicate greater levels of health insurance literacy. To facilitate interpretation of the regression results, we categorized the scaled score into 12-point intervals, as this approximated the SD and allowed us to ensure a meaningful difference in categories by classifying the full 84-point score range into 7 categories (0-12, 13-24, 25-36, 37-48, 49-60, 61-72, and 73-84).

We also report some descriptive statistics based on individuals categorized as lower HILM score vs higher HILM score. We were most interested in how participants with the lowest HILM scores compared with all others, because these would represent individuals for whom a future intervention to improve health insurance literacy would be designed. As a result, participants with HILM scores in the bottom third of the sample were designated as lower HILM (HILM scores, 0-60) and all other participants (HILM scores, 61-84) were designated as higher HILM for these basic descriptive analyses. General health literacy^32^ and numeracy^33^ were also assessed using previously validated measures. For general health literacy, a 3-question screening assessment was used. Responses were scored on a Likert scale, as previously validated, and the mean was estimated across the 3 items (score range, 1-5). Higher scores indicate greater perceived ability to understand health information. For numeracy, the 4-item ability subscale of the Subjective Numeracy Scale was used. Responses were scored on a Likert scale and the mean was estimated across the 4 items (score range, 1-6). Higher scores indicate greater perceived ability and interest in using numbers.

#### Demographic and Health Characteristics

We assessed demographics (age, sex, race/ethnicity, educational level, income, geographic location),^34^ health status,^30^ chronic conditions,^35^ insurance status,^34^ and enrollment in a high-deductible health plan^29^ (private health insurance plan with a deductible greater than $1300 for an individual or $2600 for a family), using standard items from established surveys. For income, we used the midpoint of each income category and household size to estimate participants’ income as a percentage of the federal poverty level.

### Statistical Analysis

We used descriptive statistics to report responses to individual survey items and bivariate and multivariable logistic regression to explore associations between health insurance literacy and our dependent variables (avoidance and use of preventive and nonpreventive health services). In multivariable analyses, we adjusted for age, sex, race/ethnicity, income, educational level, high-deductible health plan, health literacy, numeracy, and chronic health conditions. Adjusted odds ratios (aORs) and 95% CIs were calculated based on these multivariable regression results. Predicted probabilities were also assessed for selected examples by calculating marginal estimates based on multivariable regression results. We also conducted analyses that were stratified by whether the survey participant did or did not have a high-deductible health insurance plan, as those individuals may be responsible for more out-of-pocket costs before insurance starts sharing the costs of care. Stata, version 13 (StataCorp), was used for all analyses, and a 2-sided *P* value <.05 was considered statistically significant.

## Results

### Participant Characteristics

The survey was completed by 506 of the 511 participants who began it (participation rate, 99.0%).^36^ Participants had a mean (SD) age of 34 (10.4) years, with 339 (67.0%) younger than 35 years. A total of 228 participants (45.1%) were women, 406 were white (80.6%), 41 were Hispanic (8.1%), and 245 were college educated (48.4%), with diverse representation of income groups and geographic locations (Table 1). Almost half (228 [45.1%]) had at least 1 chronic health condition. A total of 361 of 500 participants (72.2%) who responded to the survey item had significant experience with health care (≥1 outpatient visit in the past 12 months) and 446 of the 501 participants (89.0%) who responded to the survey item had their current health insurance plan for at least 12 months.

**Table 1.** Survey Participant Characteristics

Characteristics	All Participants (N = 506), No. (%)
Age, y	
18-26	116 (22.9)
27-35	223 (44.1)
36-49	110 (21.7)
50-64	53 (10.5)
≥65	4 (0.8)
Sex	
Male	275 (54.4)
Female	228 (45.1)
Other	3 (0.6)
Race/ethnicity^a^	
White/Caucasian	406 (80.6)
Black/African American	42 (8.3)
Asian/Asian American	40 (7.9)
American Indian/Alaskan Native	12 (2.4)
Other	4 (0.8)
Hispanic	41 (8.1)
Arab	7 (1.4)
Educational level	
Less than high school	1 (0.2)
High school/GED	130 (25.7)
Vocational/2-y degree	130 (25.7)
4-y College or more	245 (48.4)
Income, % federal poverty level	
0-138	105 (21.1)
139-250	125 (25.1)
251-400	158 (31.7)
>400	110 (22.1)
Geographic location	
Northeast	108 (21.3)
Midwest	101 (20.0)
South	162 (32.0)
West	135 (26.7)
No. of chronic conditions^b^	
0	278 (54.9)
1	140 (27.7)
≥2	88 (17.4)
Type of chronic condition	
Diabetes	15 (3.0)
Hypertension	83 (16.4)
MI, CAD, or CHF	5 (1.0)
Stroke	5 (1.0)
Asthma, chronic bronchitis, COPD, or emphysema	52 (10.3)
Chronic kidney disease or dialysis	6 (1.2)
Cancer, except skin cancer	15 (3.0)
Depression or anxiety	156 (30.9)
Alcoholism or drug addiction	18 (3.6)
No. of outpatient visits	
0	139 (27.8)
1	138 (27.6)
2	122 (24.4)
≥3	101 (20.2)
Mean (SD)	2.43 (4.55)
No. of months with current insurance plan^c^	
≥12	446 (89.0)
High-deductible health plan^d^	131 (25.9)

Abbreviations: CAD, coronary artery disease; CHF, congestive heart failure; COPD, chronic obstructive pulmonary disease; GED, general education diploma; MI, myocardial infarction.

^a^ Of the 506 participants, 504 reported their race, 505 responded to the Hispanic ethnicity item, and 502 responded to the Arab ethnicity item.

^b^ Chronic condition defined as a response of yes to any of the listed conditions.

^c^ Of the 506 participants, 501 responded to the item.

^d^ High-deductible health plan is a private health insurance plan with a higher amount an individual must pay for health care costs before the insurance plan covers the cost (the deductible). This is defined as more than $1300 for an individual or $2600 for a family.

### Delayed or Foregone Care

There were 150 participants (29.6%) who reported having delayed or foregone care owing to cost in the past 12 months. For preventive care, 80 participants (15.8%) reported avoidance of preventive services, including 53 (10.5%), physical examination; 26 (5.1%), cholesterol level check; 27 (5.3%), influenza vaccination; 17 (3.4%), colon cancer screening test; 20 (4.0%), a mammogram; and 30 (5.9%), a Papanicolaou test (Table 2). For nonpreventive care, 76 participants (15.0%) reported avoidance of nonpreventive services, including 50 (9.9%), an urgent care visit; 13 (2.6%), a roentgenogram; and 40 (7.9%), magnetic resonance imaging. We also observed greater avoidance of both preventive and nonpreventive care for those in high-deductible health plans (eTable 1 in the Supplement).

**Table 2.** Health Insurance Literacy Measure and Cost-Related Delayed or Foregone Services^a^

Type of Delayed/Foregone Care	No. (%)^b^			
	Participants		HILM Score	
	All	Eligible	Lower, 1-60	Higher, 61-84
Preventive care	80 (15.8)	80 (15.8)	43 (23.8)	37 (11.4)
Physical examination	53 (10.5)	53 (10.5)	31 (17.1)	22 (6.8)
Cholesterol level check	26 (5.1)	8 (6.0)	12 (6.6)	14 (4.3)
Influenza vaccination	27 (5.3)	27 (5.3)	15 (8.3)	12 (3.7)
Colon cancer screening test	17 (3.4)	3 (5.3)	10 (5.5)	7 (2.2)
Mammogram	20 (4.0)	2 (5.7)	12 (6.6)	8 (2.5)
Papanicolaou test	30 (5.9)	26 (11.6)	17 (9.4)	13 (4.0)
Nonpreventive care	76 (15.0)	76 (15.0)	35 (19.3)	41 (12.6)
Urgent visit	50 (9.9)	50 (9.9)	24 (13.3)	26 (8.0)
Radiograph	13 (2.6)	13 (2.6)	6 (3.3)	7 (2.2)
Magnetic resonance imaging	40 (7.9)	40 (7.9)	18 (9.9)	22 (6.8)

Abbreviation: HILM, Health Insurance Literacy Measure.

^a^ A total of 150 of the 506 survey participants (30% of overall sample) had delayed or foregone care owing to cost. Summary variables for preventive care and nonpreventive care were considered yes if yes was indicated for any of the services listed below in each section. Statistical analyses for aggregated delayed or foregone preventive care and nonpreventive care outcomes are represented in Table 3.

^b^ Participant eligibility for health care services was defined as cholesterol level check (men aged ≥35 years and women aged ≥45 years; n = 133); colon cancer screening test (participants aged 50-75 years; n = 57); mammogram (women aged 21-65 years; n = 35); Papanicolaou test (women aged 21-65 years; n = 224); all other health care services (all participants; N = 506). The HILM scores dichotomized at 60 into lower HILM (n = 181) and higher HILM (n = 325). The denominator changes for each service, depending on the reporting of eligible respondents.

### Health Insurance Literacy

The mean (SD) HILM score was 63.5 (12.3) of a maximum possible score of 84 across the 21 items assessed. The HILM score was significantly positively correlated with numeracy and income (eTable 2 in the Supplement reports all bivariate correlations). The overall sample had a mean general health literacy score of 2.54 (0.57) and mean numeracy of 4.56 (1.23).

### Association of Health Insurance Literacy With Delayed or Foregone Preventive vs Nonpreventive Care

Participants with lower HILM scores had higher rates of avoiding preventive services (43 of 181 [23.8%] lower HILM vs 37 of 325 [11.4%] higher HILM groups) and nonpreventive services (35 of 181 lower HILM [19.3%] vs 41 of 325 [12.6%] higher HILM groups) (Table 2). Because of the low numbers of participants who received each service, we did not conduct statistical analyses for individual services but considered these services in aggregate for any preventive or nonpreventive care in regression analyses.

In multivariable logistic regression analyses, each 12-point increase in HILM score was associated with a lower likelihood of delayed or foregone care owing to cost for preventive care (aOR, 0.61; 95% CI, 0.48-0.78) and also for nonpreventive care (aOR, 0.71; 95% CI, 0.55-0.91) (Table 3). For example, an individual with an HILM score of 50 (approximately 1 SD below the mean) would have a 22.8% predicted probability of delaying or foregoing preventive services compared with an individual with an HILM score of 75 (approximately 1 SD above the mean), who would have a 9.9% predicted probability of delaying or foregoing preventive services owing to cost. For nonpreventive services, the lower HILM individual would also have a higher predicted probability of delaying or foregoing care (19.5%) compared with the higher HILM individual (11.4%).

**Table 3.** Association of Health Insurance Literacy With Cost-Related Delayed or Foregone Care for Selected Preventive and Nonpreventive Services^a^

Type of Delayed/Foregone Care	Health Insurance Literacy Measure			
	Unadjusted OR (95% CI)	*P* Value	Adjusted OR (95% CI)^b^	*P* Value
Preventive care	0.67 (0.54-0.83)	<.001	0.61 (0.48-0.78)	<.001
Nonpreventive care	0.79 (0.64-0.99)	.04	0.71 (0.55-0.91)	.007

Abbreviation: OR, odds ratio.

^a^ These findings represent an aggregate for delaying or foregoing any selected preventive (physical examination, cholesterol level check, influenza vaccination, colonoscopy, mammogram, Papanicolaou test) or nonpreventive (urgent visit, radiograph, magnetic resonance imaging) care. Because of the low numbers of participants who received each individual service, statistical analyses were not considered appropriate for individual items. Odds ratios represent the association between a 12-point increase in the Health Insurance Literacy Measure score and the outcomes of delayed or foregone preventive or nonpreventive care.

^b^ Adjusted for age, sex, race/ethnicity, income, educational level, having a high-deductible health plan, health literacy, numeracy, and presence of chronic conditions.

### Association of Health Insurance Literacy With Use of Preventive vs Nonpreventive Services

We also examined participants’ reported use of selected preventive (influenza vaccination, cholesterol level check) and nonpreventive (emergency department visit, hospital admission) services in the full sample of participants (eTable 3 in the Supplement). Each 12-point increase in HILM score was associated with a higher likelihood of preventive services use (aOR, 1.57; 95% CI, 1.28-1.92), but no significant change in nonpreventive services use (aOR, 1.23; 95% CI, 0.93-1.63) (Table 4). When we compared an individual with an HILM score of 50 with an individual with an HILM score of 75, the participant with the lower HILM score had a 53.1% predicted probability of any preventive services use compared with 74.8% for the participant with the higher HILM score. For nonpreventive services use, the predicted probability was more similar between individuals with a lower (12.1%) and higher (17.0%) HILM score. In sensitivity analyses for all regressions, we additionally examined the results continuously and the results did not change.

**Table 4.** Association Between Health Insurance Literacy and Preventive and Nonpreventive Service Use

Use of Health Care Services	Health Insurance Literacy Measure^a^			
	Unadjusted OR (95% CI)	*P* Value	Adjusted OR (95% CI)^b^	*P* Value
Preventive services				
Use of either preventive service below	1.57 (1.31-1.88)	<.001	1.57 (1.28-1.92)	<.001
Cholesterol level check	1.69 (1.41-2.04)	<.001	1.69 (1.38-2.07)	<.001
Influenza vaccination	1.20 (0.98-1.46)	.07	1.19 (0.96-1.48)	.11
Nonpreventive services				
Use of either nonpreventive service below	1.11 (0.87-1.42)	.41	1.23 (0.93-1.63)	.14
ED visit	1.08 (0.84–1.40)	.55	1.22 (0.91-1.63)	.18
Hospital admission	1.39 (0.87-2.22)	.17	1.50 (0.91-2.46)	.11

Abbreviations: ED, emergency department; OR, odds ratio.

^a^ Odds ratios represent the change in odds with each 12-point increase in Health Insurance Literacy Measure, with higher scores indicating greater health insurance literacy.

^b^ Adjusted for age, sex, race/ethnicity, income, educational level, having a high-deductible health plan, health literacy, numeracy, and presence of chronic conditions.

## Discussion

In this national study of an insured sample, 150 people (29.6%) reported delaying or foregoing health care owing to perceptions of costs. Although participants were overall equally likely to avoid preventive and nonpreventive care owing to cost concerns, those with lower health insurance literacy reported significantly greater avoidance of both preventive and nonpreventive services. Likewise, participants with lower health insurance literacy were less likely to report use of preventive services. These findings suggest that health insurance literacy is important for patients, not only while selecting a health plan, but also in health care navigation and uptake of recommended health services.

There are several possible explanations for the study’s findings. First, it is likely that individuals with lower health insurance literacy may not understand many cost-sharing and cost-reduction features of their health plan, despite the ACA mandate that preventive services be covered without out-of-pocket costs to consumers. Thus, they may be less likely to use care they perceive as optional, particularly preventive care. Second, patients may perceive other costs besides monetary copayments, such as taking time off from work, arranging for child care, and waiting during long medical appointments. Such costs would not be addressed by the copayment and deductible exemption. Because these nonmonetary costs may be felt more by individuals of lower socioeconomic status, we adjusted for income and educational level and found no change in the study results. Third, it is possible that those with greater health insurance literacy generally were more knowledgeable about the value of preventive services. However, our findings were consistent even after adjustment for general health literacy and numeracy, which could potentially be associated with knowledge about the value of preventive services.

Our findings echo those of earlier research examining the importance of general health literacy to health care access. Levy and Janke^37^ found that individuals with lower general health literacy were more likely to delay care or have difficulty accessing health care than individuals with adequate health literacy. Our study suggests a similar association between patients’ health insurance literacy and their ability to navigate and receive health care.

This study may have implications for policy and program changes that aim to improve communication about health insurance policies and patients’ health care decision making. Furtado and colleagues^38^ noted that uninsured individuals’ most trusted sources of health insurance information were health care professionals and their social networks. Thus, developing simple messages that can be delivered by health plans, health care professionals, community health workers, or health navigators may help to make health insurance concepts accessible and trusted by patients. Plain-language communication by trusted sources has the potential to significantly improve patients’ health insurance literacy and confidence making health care decisions. Community-based education may be particularly important for the newly insured and could be linked to local and national outreach and enrollment efforts.

Regardless of patients’ health insurance literacy, these messages should aim to increase their understanding that recommended preventive services are exempt from out-of-pocket costs. Simple advertising may be used by clinicians’ practices and pharmacies to draw attention to such services. In addition, health care professionals could address costs in discussions of health care recommendations.^39,40^

### Limitations

This study should be interpreted in the context of its limitations. First, although the sample was a national group of participants with economic and geographic diversity, it was not a nationally representative sample. The participants were younger and had higher educational attainment than the general population, which is a known limitation of MTurk samples.^41^ Younger age and higher educational level could be associated with greater health insurance literacy, yet many of these individuals avoided preventive health care because of perceived costs. In addition, older adults may face even greater challenges navigating health care services owing to lower health insurance literacy and greater health care needs.^3^ Second, the HILM measures confidence in understanding and using health insurance, but it does not directly measure knowledge of specific health insurance concepts. We chose to use HILM for our independent variable as, to our knowledge, it is currently the only validated measure of health insurance literacy available. Third, we cannot definitively determine whether individuals who reported delayed or foregone care would have had a medical need for the health care service. Our analyses considering the full sample in the eligible denominator may underestimate small effects of health insurance literacy on delays of specific types of health care services.

## Conclusions

To our knowledge, this study presents some of the first evidence on the importance of considering health insurance literacy in relation to health care navigation and use. We found that patients with lower health insurance literacy had greater avoidance of both nonpreventive and preventive services, despite the ACA cost-sharing exemption for recommended preventive services. Future work should examine potential drivers of foregone care among those with lower health insurance literacy as potential targets for intervention. To improve appropriate use of recommended health care services, including preventive health services, clinicians, health plans, and policymakers should adopt communication strategies that make health insurance concepts accessible to individuals regardless of health insurance literacy and improve consumers’ understanding of services exempt from out-of-pocket costs.

## Notes

Supplement. **eFigure.** Health Insurance Literacy Measure (HILM) Distribution **eTable 1.** Prevalence of Delayed or Foregone Services Due to Costs, Participants With and Without High-Deductible Health Plans **eTable 2.** Bivariate Correlations Between HILM and Demographic and Health Characteristics **eTable 3.** Health Care Utilization Among Survey Participants, Preventive and Non-Preventive Services
