# Supplementary material for: Association Between Health Insurance Literacy and Avoidance of Health Care Services Owing to Cost
Source: JAMA Netw Open. 2018 Nov 16;1(7):e184796. doi: 10.1001/jamanetworkopen.2018.4796 (PMC6324372; doi:10.1001/jamanetworkopen.2018.4796)
Supplement: Supplement. — eFigure. Health Insurance Literacy Measure (HILM) Distribution eTable 1. Prevalence of Delayed or Foregone Services Due to Costs, Participants With and Without High-Deductible Health Plans eTable 2. Bivariate Correlations Between HILM and Demographic and Health Characteristics eTable 3. Health Care Utilization Among Survey Participants, Preventive and Non-Preventive Services [file jamanetwopen-1-e184796-s001.pdf]

Supplementary Online Content

Tipirneni R, Politi MC, Kullgren JT, Kieffer EC, Goold SD, Scherer AM. Association between health insurance literacy and avoidance of health care services owing to cost. *JAMA Netw Open*. 2018;1(7):e184796. doi:10.1001/jamanetworkopen.2018.4796

eFigure. Health Insurance Literacy Measure (HILM) Distribution

eTable 1. Prevalence of Delayed or Foregone Services Due to Costs, Participants With and Without High-Deductible Health Plans

eTable 2. Bivariate Correlations Between HILM and Demographic and Health Characteristics

eTable 3. Health Care Utilization Among Survey Participants, Preventive and Non-Preventive Services

This supplementary material has been provided by the authors to give readers additional information about their work.

.

© 2018 Tipirneni R et al. *JAMA Network Open.*

eFigure. Health Insurance Literacy Measure (HILM) Distribution

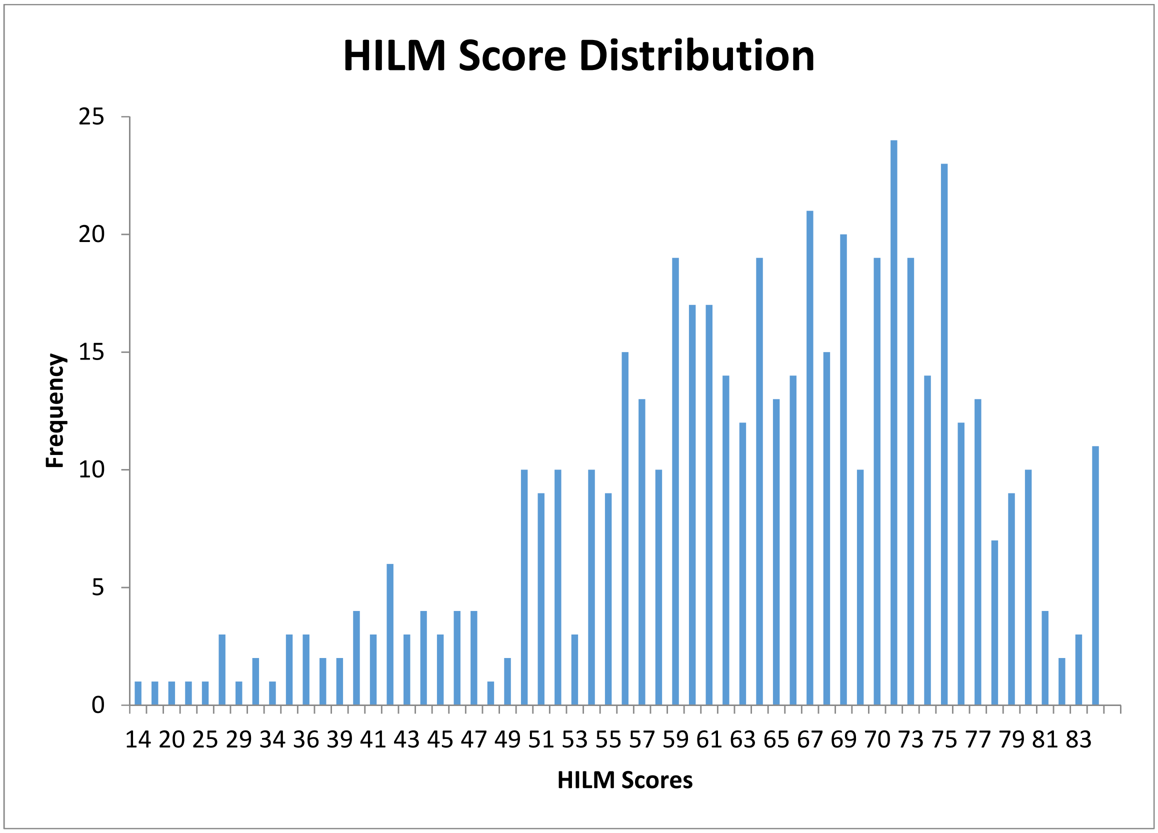

© 2018 Tipirneni R et al. *JAMA Network Open.*

eTable 1. Prevalence of Delayed or Foregone Services Due to Costs, Participants With and Without High-Deductible Health Plans[a]

	Delayed/Foregone Care, n (%)			
**Type of Care**	**All Participants (N=506)**	**HDHP[b] (n=131)**	**Non-HDHP[b] (n=375)**	***p* value**
Delayed/Foregone Preventive Care	**80 (15.8)**	32 (24.4)	48 (12.8)	0.003
Physical	53 (10.5)	22 (16.8)	31 (8.3)	
Cholesterol Check	26 (5.1)	11 (8.4)	15 (4.0)	
Flu Shot	27 (5.3)	9 (6.9)	18 (4.8)	
Colon cancer screening	17 (3.4)	10 (7.6)	7	
Mammogram	20 (4.0)	10 (7.6)	10 (2.7)	
Pap Smear	30 (5.9)	14 (10.7)	16 (4.3)	
Delayed/Foregone Non-Preventive Care	**76 (15.0)**	32 (24.4)	44 (11.7)	0.001
Urgent visit	50 (9.9)	25 (19.1)	25 (6.7)	
X-ray	13 (2.6)	6 (4.6)	7	
MRI	40 (7.9)	17 (13.0)	23 (6.1)	

Note: [a]150 (30% of sample) had delayed or foregone care due to cost.

[b]HDHP = High Deductible Health Plan, a type of health insurance plan with a higher amount an individual must pay for health care costs before the insurance plan covers the cost (the deductible). This is defined as more than $1,300 for an individual or $2,600 for a family.

eTable 2. Bivariate Correlations Between HILM and Demographic and Health Characteristics[a]

	Health insurance literacy	Health literacy	Numeracy	Age	Gender, female	Race, non-white	Education	Income	HDHP	Chronic conditions
Health insurance literacy	-									
Health literacy	0.04 (0.327)	-								
Numeracy	0.19 (<0.001)	-0.07 (0.128)	-							
Age	0.07 (0.093)	-0.13 (0.004)	0.11 (0.018)	-						
Gender, female	0.02 (0.610)	0.04 (0.389)	-0.17 (<0.001)	0.13 (0.004)	-					
Race, non-white	-0.03 (0.523)	-0.05 (0.275)	-0.05 (0.304)	-0.07 (0.124)	-0.03 (0.486)	-				
Education	0.06 (0.215)	-0.02 (0.708)	0.28 (<0.001)	0.04 (0.413)	0.01 (0.856)	0.04 (0.366)	-			
Income	0.14	-0.01	0.19	0.07	-0.04	-0.01	0.32	-		

© 2018 Tipirneni R et al. *JAMA Network Open.*

	(0.001)	(0.982)	(<0.001)	(0.100)	(0.373)	(0.807)	(<0.001)			
HDHP	0.05<br><br>(0.310)	0.05<br><br>(0.255)	0.01<br><br>(0.937)	0.01<br><br>(0.917)	0.01<br><br>(0.778)	-0.03<br><br>(0.469)	0.12<br><br>(0.006)	0.24<br><br>(<0.001)	-	
Chronic conditions	-0.02<br><br>(0.591)	0.07<br><br>(0.097)	-0.09<br><br>(0.053)	0.12<br><br>(0.009)	0.06<br><br>(0.186)	-0.12<br><br>(0.005)	-0.13<br><br>(0.004)	-0.14<br><br>(0.002)	0.02<br><br>(0.688)	-

Note: [a]Correlations and *p* values (in parentheses). Cells in grey indicate $p<0.05$. HILM = Health Insurance Literacy Measure; HDHP=High Deductible Health Plan.

© 2018 Tipirneni R et al. *JAMA Network Open.*

eTable 3. Health Care Utilization Among Survey Participants, Preventive and Non-Preventive Services

Health care service in the past 12 months	N (%)
**Preventive Services**	
Flu shot in the past 12 months	135 (26.7)
Cholesterol check in the past 5 years	301 (59.6)
Any preventive service use (flu shot or cholesterol check)	326 (64.6)
**Non-Preventive Services**	
Emergency Room visits (mean 0.18, SD 0.54 visits)	
0	441 (87.2)
≥1	65 (12.8)
Hospital Admissions (mean 0.05, SD 0.24 visits)	
0	483 (95.6)
≥1	22 (4.4)
Any non-preventive service use (ER or inpatient)	73 (14.5)

© 2018 Tipirneni, R, et al. *JAMA Network Open.*
